# Lack of Support for the Association between Facial Shape and Aggression: A Reappraisal Based on a Worldwide Population Genetics Perspective

**DOI:** 10.1371/journal.pone.0052317

**Published:** 2013-01-09

**Authors:** Jorge Gómez-Valdés, Tábita Hünemeier, Mirsha Quinto-Sánchez, Carolina Paschetta, Soledad de Azevedo, Marina F. González, Neus Martínez-Abadías, Mireia Esparza, Héctor M. Pucciarelli, Francisco M. Salzano, Claiton H. D. Bau, Maria Cátira Bortolini, Rolando González-José

**Affiliations:** 1 Departamento de Anatomía, Facultad de Medicina, Universidad Nacional Autónoma de México, Distrito Federal, México; 2 Departamento de Genética, Instituto de Biociências, Universidade Federal do Rio Grande do Sul, Porto Alegre, Rio Grande do Sul, Brazil; 3 Centro Nacional Patagónico, Consejo Nacional de Investigaciones Científicas y Técnicas, Puerto Madryn, Argentina; 4 EMBL-CRG Systems Biology Research Unit, Center for Genomic Regulation, Universitat Pompeu Fabra, Barcelona, Spain; 5 Secció d'Antropologia, Departament de Biologia Animal, Universitat de Barcelona, Barcelona, Spain; 6 División Antropología del Museo de La Plata, Consejo Nacional de Investigaciones Científicas y Técnicas, La Plata, Argentina; University of Cambridge, United Kingdom

## Abstract

Antisocial and criminal behaviors are multifactorial traits whose interpretation relies on multiple disciplines. Since these interpretations may have social, moral and legal implications, a constant review of the evidence is necessary before any scientific claim is considered as truth. A recent study proposed that men with wider faces relative to facial height (fWHR) are more likely to develop unethical behaviour mediated by a psychological sense of power. This research was based on reports suggesting that sexual dimorphism and selection would be responsible for a correlation between fWHR and aggression. Here we show that 4,960 individuals from 94 modern human populations belonging to a vast array of genetic and cultural contexts do not display significant amounts of fWHR sexual dimorphism. Further analyses using populations with associated ethnographical records as well as samples of male prisoners of the Mexico City Federal Penitentiary condemned by crimes of variable level of inter-personal aggression (homicide, robbery, and minor faults) did not show significant evidence, suggesting that populations/individuals with higher levels of bellicosity, aggressive behaviour, or power-mediated behaviour display greater fWHR. Finally, a regression analysis of fWHR on individual's fitness showed no significant correlation between this facial trait and reproductive success. Overall, our results suggest that facial attributes are poor predictors of aggressive behaviour, or at least, that sexual selection was weak enough to leave a signal on patterns of between- and within-sex and population facial variation.

## Introduction

Since the work published by Gall in 1835 [1], there has been a persistent interest in exploring methods aimed to predict the behavioural, moral, ethic, or emotional status of an individual departing from their physical appearance in general, and from their craniofacial shape in particular. Under Gall's perspective, an individual's moral and intellectual *faculties* are innate and determined by the brain *organs* (e.g. size and shape of the brain). Since the form of the head is a good predictor of brain shape, it follows from this perspective that an individual's morality could be predicted by its head shape. This research program attained its maximum splendour during the mid-19th century under the label of phrenology, and was revitalized during early 20th century positivism as an attempt to solve criminological cases. The most prominent defender of this school was Cesare Lombroso, who argued that criminals are distinguished from non-criminals by a set of physical anomalies, reminiscent of primitive, ancestral human stages [2].

The advent of the population genetics paradigm provided a new scenario for the analysis of human evolutionary and developmental patterns of craniofacial variation. Along with research on the socio-cultural variability of human behaviours performed from the last half of the 20th century, it has been demonstrated that there is no straightforward connection between behaviour and physical appearance [3]. Even though changes on behaviour likely facilitated the evolutionary success of early hominines, behaviour is a very complex and plastic phenotype that can be quickly reshaped through education and other socio-cultural practices [4]–[8]. In addition, it has been shown that neuronal and brain functions are particularly amenable to plasticity [9].

However, a handful of recent articles [10]–[15] have challenged this view and suggested that simple facial traits can be used to predict aggressive, unethical and other kind of behaviours. Particularly, one of these papers [10] reported that men with higher fWHR scores (facial width-to-height ratio) are more likely to develop unethical behaviour mediated by a psychological sense of power. According to these authors, men with greater fWHR feel more powerful, which directly leads to less ethical behaviour, including lying and cheating [10]. This research was based on reports suggesting that sexual dimorphism and selection would be responsible for a correlation between fWHR and aggression [11]–[15]. Since aggression has been positively associated with mate preference [10] and unethical behaviour, positive selection for fWHR should be then regarded as a reliable signal of male dominance. If this preference for males with greater fWHR scores occurred ubiquitously during modern human evolution, an expected outcome is a worldwide high level of sexual dimorphism for fWHR, with greater values of fWHR being displayed by men coming from populations with high levels of inter-personal aggression. Another expected outcome is a positive correlation among masculine fitness and fWHR, indicating that men with higher fWHR have higher reproductive success.

Some recent papers have demonstrated that fWHR is not dimorphic on a sample of Turkish university students [16], as well as on samples of “Europeans” and “Africans” [17]. Also, some recent papers demonstrated that fWHR is not associated with self-reported aggression [16], or with indirect measurements of aggression (hockey penalties) [18]. In addition, a recent paper by Kramer et al. [19] using large, typologically-labeled samples (e.g. “white german, white british”) found no evidence of sexual dimorphism on fWHR. Furthermore, Stirrat and Perrett [20] demonstrated that men with greater fWHRs were perceived as less attractive (running counter to a “mate preference” explanation), and Stirrat et al [21] found that men with smaller fWHRs were more likely than men with greater fWHRs to die from male-male physical violence.

Even when the papers detailed above present contradictory evidence regarding the alleged adaptive nature of fWHR variation, it is still necessary to test this adaptive hypothesis on worldwide, cross-cultural populations, to measure fWHR sexual dimorphism on groups displaying differing levels of interpersonal violence, and to evaluate putative associations among male fitness and fWHR. Cross-cultural work, particularly in traditional societies, will be especially useful to this end [22]. Therefore, the objectives of this work are to further test fWHR dimorphism on a population based, world-wide level, to evaluate the null prediction of an association among aggression levels and fWHR on a broader quantitative and population-genetic context, and to verify if males with greater fWHR present higher fitness values. Our sampling strategy is thus aimed to maximize cultural, economic, linguistic and geographic coverage and included seven different, previously published databases covering 4,960 individuals from 94 modern worldwide populations. To sum up, the framing of our paper is exclusively on testing the theoretical expectations derived from the adaptive explanations previously stated (especially in references 10–14) on a global, cross-cultural sample. Note that both, intra and intersexual selection were suggested to explain a putative adaptive role of fWHR, and that both scenarios predict significant male dimorphism if adaptation effectively occurred. In a revision of sexual selection mechanism in humans, Puts [22] profusely showed that ancestral selection pressures can be inferred by studying the adaptations that they produced. A condition for selective processes to be demonstrated (or at least supported by evidence taken on natural populations) is that changes in allelic frequencies of the genes underlying the expression of the selected phenotypes vary from one generation to the next. If these conditions are not met, adaptation is just one of many alternative explanations concerning the studied phenomenon.

## Materials and Methods

### The sample

To maximize the number of sampled populations and to achieve large sample sizes, we based our analysis on previously published databases that involve several types of raw data, including craniofacial measurements, 2D and 3D craniofacial landmark coordinates taken on dried skulls. We verified that for each database a good estimator of fWHR could be computed. Note that all of these methods to estimate fWHR are superior to that based on non oriented photographs [10], [12], [13], [18], [20], where non-controlling of coplanarity introduces potential measurement errors that may arise from artefacts of head posture, i.e. faces rotated with respect to the camera in the horizontal or vertical planes [17], [19].

Databases were analyzed separately because of subtle differences in measurement or landmark definitions. Nevertheless, all the databases matched with the general definition of fWHR (the ratio of bizygomatic breadth to nasion-prostion height). Sample composition and specific details of each database are provided on Table 1, whereas ethnographic information is provided in Table S1. Note that two databases (México and Hallstat) were used for specific purposes: the Mexican database was used to test differences among groups differing on levels of interpersonal violence, whereas the Hallstatt database was used to estimate the correlation among fWHR scores and male fitness.

**Table 1:** Databases used in this study. Population lists for each database are provided in Table S1.

Database	Type of Data	References	Number of populations	Number of raw measurements	Sample Size (female/male)	Indices computed	Average (min-max) Dimorphism^1^	Average WHR Dimorphism and associated p value^2^	Average (min-max) male Fst^3^	Male Fst WHR
Howells	Cranial measurements	[39]–[40]	26	71	2,412 (1,156/2,256)	WHR, CI, CHLI, CBHI, CTI, GI, JFI, OI, NI, GP	1.048 (0.0872–2.265)	1.006 (0.076)	0.281 (0.166–0.400)	0.166
Pucciarelli	Cranial measurements	[41]–[43]	23	30	440 (143/297)	WHR, CI, CHLI, TI, OI, NI, NVI, FVI, ANVI, MNVI, PNVI, OTVI, OVI, RVI, MVI, AVI, NFI, ANMI, MNMI, PNMI, TMI, OMI, RMI, MMI, AMI	1.038 (0.614–1.916)	1.016 (0.080)	0.173 (0.122–0.296)	0.126
2D	Two dimensional craniofacial landmark coordinates	[44]–[46]	19	n/a	580 (278/302)	WHR, CI, CHLI, CBHI, NI	1.016 (0.838–1.157)	1.020 (0.082)	0.199 (0.134–0.307)	0.134
3D	Three dimensional craniofacial landmark coordinates	[47]–[48]	13	n/a	782 (381/401)	WHR, CI, CHLI, CBHI, CTI, GI, NI, GP	1.002 (0.994–1.009)	1.004 (0.970)	0.223 (0.042–0.408)	0.078
Patagonia	Cranial measurements	[49]–[50]	8	28	260 (149/111)	WHR, CI, CHLI, CBHI, CTI, GI, JFI, OI, NI	1.021 (0.860–1.383)	1.002 (0.731)	0.057 (0.009–0.164)	0.014
Mexico City Penitentiary and general population	Cranial measurements	[26]	4	23	163 (0/163)	WHR	n/a	n/a	0.014 (0.000–0.074)	0.074
Hallstat	Three dimensional craniofacial landmark coordinates	[27]–[28]	1	n/a	296 (117/179)	WHR	1.001 (0.956–1.038)	1.038 (0.002)	n/a	n/a

1 : computed across all indices and across all populations.

2 : computed across all populations.

3 : computed across all indices. Indices definitions are as follows: WHR: Width-to heigth ratio, bizygomatic breadth/nasion-prosthion height; CI: Cephalic index, maximum cranial breadth ×100/maximum cranial length; CHLI: Cranial height-length, height (from bregma to basion or porion) ×100/maximum cranial length; CBHI: Cranial breadth-height, maximum cranial height ×100/maximum cranial breadth; CTI: Craniofacial transverse index, bizygomatic breadth ×100/maximum cranial breadth; GI: Gnathic index, basion-prosthion length ×100/basion-nasion length; JFI: Jugofrontal index, minimum frontal breadth ×100/bizygomatic breadth; OI: Orbital index: maximum orbital height ×100/maximum orbital breadth; NI: Nasal index: maximum nasal breadth ×100/nasal height; GP: Glabellar projection index, glabella-opisthocranion length/nasionopisthocranion length; NVI: Neurocranial volumetric index; FVI: Facial volumetric index; NFI: Neurofacial index; ANVI: Anteroneural volumetric index; MNVI: Midneural volumetric index; PNVI: Posteroneural volumetric index; OTVI: Otic volumetric index; OVI: Optic volumetric index; RVI: Respiratory volumetric index; MVI: Masticatory volumetric index; AVI: Alveolar volumetric index; ANMI: Anteroneural morphometric index; MNMI: Midneural morphometric index; PNMI: Posteroneural morphometric index; OTMI: Otic morphometric index; OMI: Optic morphometric index; RMI: Respiratory morphometric index; MMI: Masticatory morphometric index; AMI: Alveolar morphometric.

For each database, a variable amount of indices were computed according to traditional formulae (see Table 1). These indices depict general aspects of skull shape as well as localized structures (nasal, orbital, alveolar, etc.).

### Evaluation of fWHR sexual dimorphism at global and population levels

A global estimation of sexual dimorphism was computed as the ratio among the male to the female average value for each index. Statistical significance of sexual dimorphism for each index on each database was evaluated by t-tests for independent samples. Then, the significance of sexual dimorphism was evaluated at the population level, within each database. To analyze the apportionment of population variation across the different indices, male and female Fst values for each index were also computed [23]. The fixation index, Fst, is a measure of the proportion of diversity due to differences among populations [23].

### Evaluation of fWHR sexual dimorphism on groups displaying differing levels of interpersonal violence

Specific subsets of populations were selected to compare fWHR's sexual dimorphism on populations for which ethnographic records of within-group interpersonal violence [24] are available. First, we compared differences on among-population sexual dimorphism on worldwide populations holding well documented ethnographic records of within-group inter-personal violence levels along with presence of fighting games or rituals [25]. Ethnographers and other scholars have estimated the lethal violence level as an aspect of the general pattern of inter/intra-group aggression or conflict in modern and ancient human societies. Percentage of deaths in warfare and homicide rates are the most frequently used quantitative indices. In a recent compilation, Pinker [24] showed that the average percentage of death in warfare considering simple nomadic hunter-gatherer and forager-horticulturalist prestate societies are 12% and 22%, respectively, while for state societies the percentages are normally lower. In consequence, the different social organization systems present contrasting patterns of violence and sociality in very broad terms. Thus, we have further classified, when possible, populations within each database as hunter-gatherers (HG), farmers (F) and state society (SS) in order to test if fWHR sexual dimorphism is higher on society types displaying higher levels of interpersonal violence. Comparisons were limited to the Howells, Pucciarelli, 2D, 3D and Mexico databases, while the Patagonian dataset was not included in this analysis because it is exclusively represented by nomadic hunter-gatherer groups.

To further assess differences among groups with varying levels of interpersonal violence we performed an additional analysis based on the Mexico database, directly testing for differences on fWHR scores between samples of male prisoners of the Mexico City Federal Penitentiary condemned by crimes of variable level of inter-personal aggression (homicide, robbery, and minor faults) and a comparative sample of random, non-condemned individuals belonging to the same urban population [26].

### Computation of fitness on the Hallstat sample

Finally, we regressed and correlated male fWHR scores on a measure of reproductive fitness using a pedigreed sample from the Austrian population of Hallstatt, which furnishes a unique chance to compute quantitative genetic parameters for skull shape [27], [28]. Skulls from the Hallstatt collection are individually identified and church records can be used to reconstruct genealogical relationships, as well as to compute individual reproductive success measurements. To estimate fitness measures we reconstructed the genealogies of the Hallstatt population from the complete parish records of births, marriages and deaths from 1602 to 1900, which included 18,134 individuals. We only included those individuals with complete individual life histories, who married at least once and who survived to adulthood and reproduction (N = 2,549). We estimated fitness as lifetime reproductive success (LRS, number of children produced and raised to 15, that is to adulthood). Corresponding fWHR values were available for 179 males.

## Results

**Figure 1:**
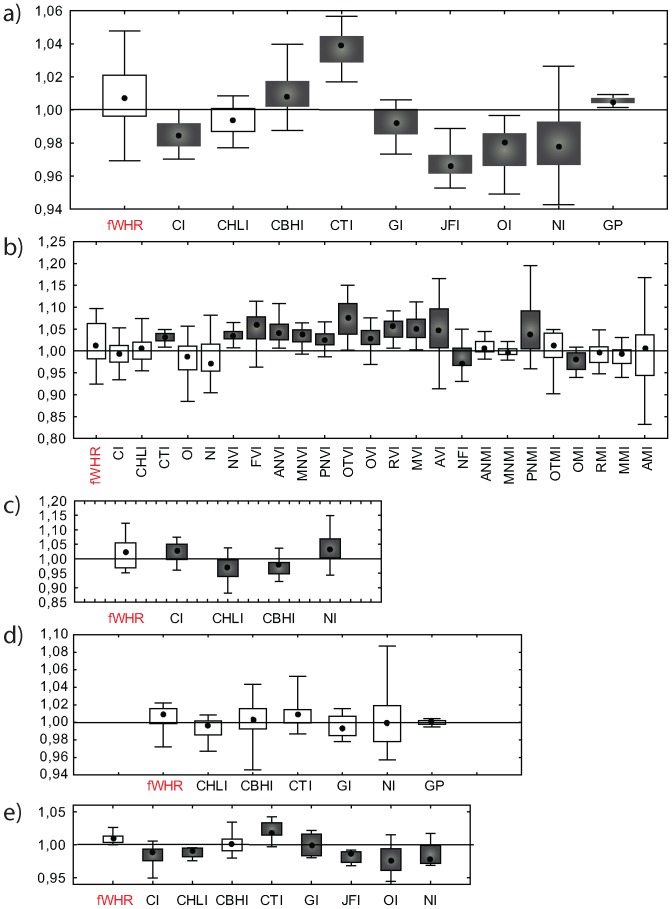
Sexual dimporphism on fWHR and further cranial indices. Box and whisker plots of global sexual dimorphism computed across the different databases. Indices that differed significantly among sexes (after t-test for independent samples) are shown in solid grey. A) Howells database; b) Pucciarelli database, c) 2D Geometric Morphometric database, d) 3D Geometric Morphometric database, e) Patagonian groups database. Square: median; box: 25%–75%; whisker: minimum-maximum values.

Our results concerning sexual dimorphism on fWHR and further indices on a worldwide scale are presented in Table 1 and Figure 1. In addition, a population-specific comparison of fWHR sexual dimorphism on each database is presented in Fig. S1. Figure 1 and Table 1 results clearly show that fWHR is among the less dimorphic indices on all the databases, and that sexual differences are not significant at *P* = 0.05, excepting for the Hallstat sample, showing significant dimorphism. Furthermore, only seven out of 89 comparisons (7.86%) yielded fWHR values significantly greater in males than in females (Figure S1). Note that all the significant comparisons became non-significant when the Bonferroni's correction is applied. The overall low Fst values obtained for fWHR (0.014–0.166) in comparison with other indices (Table 1) indicate that the pattern of within versus between-group variation is similar to estimates based on neutral DNA, protein, enzyme, and blood-group polymorphisms [29], rather than what is expected to a marker subjected to strong sexual selection.

**Figure 2:**
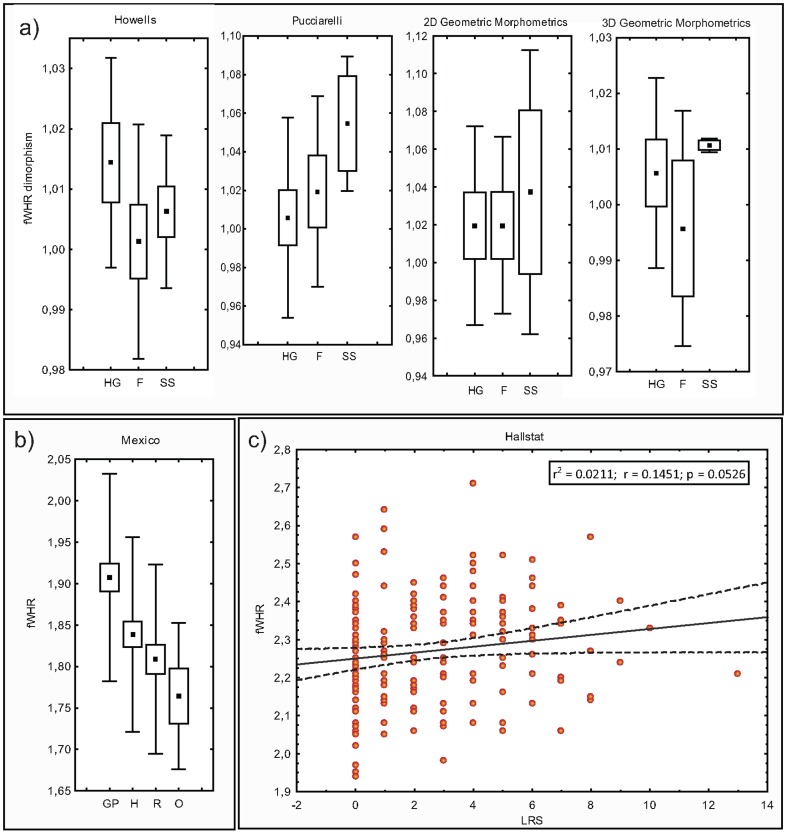
Sexual dimporphism on fWHR across socio-cultural categories. Box and whisker plots of a) fWHR sexual dimorphism in samples belonging to three different socio-cultural categories: HG: hunter-gatherers; F: farmers; SS: state societies. b) fWHR values of males from the Mexican general population (GP), males prosecuted by homicide (H), robbery (R) and other minor faults (O). Square: mean; box: standard error; whisker: standard deviation. c) Regression of fWHR on fitness, estimated as lifetime reproductive success (LRS, number of children raised to adulthood).

Results concerning fWHR dimorphism comparisons on groups with ethnographically documented variable levels of within-group interpersonal violence, and fWHR comparison in individuals subjected to prosecution decisions based on different levels of violence with non-prosecuted individuals of the same population are presented in Figure 2a and b respectively. Our results indicate no tendency of hunter-gatherers and/or forager-horticulturalists to develop greater fWHR dimorphism (Figure 2a). This suggests that this trait was not selected in males in societies were aggressive behaviour can be displayed with minimal restrictions. A more direct comparison performed on the Mexico database demonstrated that fWHR is not significantly greater on those males subjected to prosecution decisions involving crimes of variable level of inter-personal aggression in comparison with the general population (Figure 2b).

Finally, regression of fWHR on fitness estimated on the Hallstat database as lifetime reproductive success (LRS, number of children produced and raised to adulthood) [30], [31] yielded non-significant correlation between fWHR and male fitness in this population (Figure 2c). Also, note that variation on fWHR explains only a 0.0211% of the variation on fitness.

## Discussion

Previous studies assessing the relationship between fWHR and aggressiveness suggest that fWHR itself is not responsible for the behavioural phenotype, but a co-variable of another trait, such as testosterone level [32], [33]. Regardless of this relevant but often disregarded aspect, the consensus view is that behaviours normally associated with aggressiveness and its derivations, such as unethical behaviour, are more prominent in men than in women. Sexual selection pressures for traits promoting success in physical conflict, which was particularly important in ancestral environments, could bring benefits to a man as an ally or mate and may explain the predominance of aggressive behaviour in males [34]. Then, men's persistent physical traits could only predict immoral actions if they were also associated with sexual selection.

However, a growing number of studies demonstrate that fWHR is not a dimorphic trait [16], [17], [19], and that fWHR is unrelated to aggression [16], [18]. Our work expands these previous works in several aspects. First, our sample sizes are larger than previous studies thus guarantying higher statistical power, and hence more reliable results concerning sexual dimorphism. Second, we adopted a worldwide sampling strategy, focusing on geographically and culturally restricted populations, rather than on large continental/racial groupings (e.g. Caucasian, African, etc.). Third, both the scope of our paper as well as the analyses performed were strictly based on the Population Genetics expectations regarding quantitative traits subjected to sexual selection. Fourth, we provide a broader approach to craniofacial shape by including a larger number of indices, whose behavior in terms of sexual dimorphism could potentially stimulate future studies. Fifth, we have combined our cross-cultural sampling with a thoughtful review of ethnographic records contributing to a formal model of interpersonal violence developed by Pinker [24], in order to test the null hypothesis of greater fWHR sexual dimorphism on more aggressive contexts. Finally, we report a statistical evaluation of the association among fitness and fWHR, which serves as a more direct and powerful evaluation of past sexual selection.

Our results demonstrate that fWHR is a poor predictor of aggressive behaviour, or, at least, that sexual selection was weak enough to leave a signal on patterns of between and within sex and population human facial variation. More complex models of sexual selection [30], [31] might be tested to explore whether patterns of fWHR variation fit further models of positive selection favouring males with higher fWHR values (or indirectly a correlated trait such as higher levels of testosterone), but our results show that under the simplest scenario of sexual selection, the predictions of significant sexual dimorphism on fWHR (i.e., more pronounced sexual dimorphism on societies exhibiting greater levels of interpersonal violence, and greater fitness on males displaying greater fWHR values) are not corroborated by our worldwide analyses.

Our further results concerning fWHR dimorphism comparisons on groups with ethnographically documented variable levels of within-group interpersonal violence, and fWHR comparison in individuals subjected to prosecution decisions based on different levels of violence with non-prosecuted individuals also underscore that fWHR is not significantly associated with aggressive behaviour. Despite caution is needed with general classifications of society types and corresponding estimations of interpersonal violence, this type of information can be used to test whether and how fWHR varies in populations with variable influence of the socio-cultural factors controlling and preventing the more extreme forms of violence [35]. If males displaying greater fWHR scores achieved better fitness values, it would trigger a process of sexual selection focused on fWHR, and then fWHR should be higher on groups where inter personal violence is not buffered by social rules. However, our results do not support this view. Our analysis shows that there is no statistical significant association between fWHR and male fitness, even when significant sexual dimorphism is observed in the Hallstat population. To the best of our knowledge, this is the first report of a statistical evaluation of the association among fitness and fWHR, which is one of the most direct evidence of past inter and intra sexual selection.

### Evolutionary and social implications of fWHR assessment

To fully understand the inherent variation of fWHR in human populations, it must be recalled that fWHR is an attribute of the skull, which is a complex structure with a pervasive pattern of morphological integration that constraints its evolution along lines or planes of least evolutionary resistance [36]. Therefore, adaptive hypotheses regarding a specific trait like a simple facial index cannot be tested without taking into account the correlated response of genetically, epigenetically, developmentally and functionally integrated traits. Recent quantitative genetics research [28] demonstrates that the genetic plane of least evolutionary resistance for the Hallstatt population is shaped by a retraction of the lower face, an expansion of the anterior cranial vault, a forward and upward rotation of the foramen magnum, as well as a flexion of the skull about the anterior basicranium. It is likely that these constrains operated worldwide at the species level, since covariation patterns are highly conserved on modern humans [37]. None of the constraints detailed above involves the relationship among facial width and height, thus suggesting that if fWHR was indeed subjected to sexual selection, this selection force was not strong enough to operate on directions deflecting the lines of least evolutionary resistance. In other words, patterns of genetic covariation do not predict straightforward evolutionary changes involving variation on fWHR.

To sum up, we argue that, even when localized, low-scale studies may suggest that fWHR is a trait shaped by sexual selection, a population genetics approach to fWHR variation on worldwide cross-cultural modern human populations clearly show that this trait does not present any of the expected signals of past sexual selection operating during the course of human evolution. A likely explanation for these results is that behavioural repertoires are so plastic that socio-cultural environments [4]–[8] or even casual, chance associations related to small sample sizes are possibilities which, if not properly considered, may become important misleading factors in this type of analysis. On a recent paper, for instance, Wong et al. [38] found that fWHR of CEOs was positively related to the financial success of the company, but only for companies for whom the CEO used a cognitively simple leadership style. On a,

These kinds of results are at odds with adaptive explanations, and demonstrate how socio-cultural aspects are determinants of great amounts of the behavioural repertoire.

Furthermore, we suggest that analyses made on particular, highly localized samples and focused on the correlation among physical attributes and specific behaviours should incorporate and control for specific measurements of socio-cultural context in order to provide a more realistic approach to the complexity of behavioural manifestations. For instance, analyses made on different populations [16]–[19] contradicted the main expectations of the adaptive hypothesis published in references 10–15, demonstrating that fWHR is not sexually dimorphic and is not related with aggressive behaviour [16]. Finally, another important remark to studies of fWHR and aggressive behaviour is that all of them depart from the assumption that the perceiver ratings of propensity for aggression are based on the observation of neutral faces, in some cases during an exposition of few milliseconds. However, basic information is needed about how the perceiver ratings vary in more realistic conditions, including exposition to non neutral faces. For instance, fWHR decreases when smiling, since this expression generates a lateral expansion of the cheeks, and hence a “non-deterministic”, free-will drive diminution of fWHR.

Since the alleged existence of significant, statistically demonstrated and scientifically based relationship among facial attributes and behavioural traits involving morality and ethics can have social effects (e.g. prosecution decisions, work policies, police operations), we suggest that future analyses aimed to detect relationships among facial attributes and behaviour must be reinforced by cross-cultural controls, longitudinal samples, and a solid background on population genetics.

## Supporting Information

Figure S1 **Box and whisker plots of fWHR depicting inter-sexual differences across the populations of each database.** a) Howells database; b) Pucciarelli database, c) 2D Geometric Morphometric database, d) 3D Geometric Morphometric database, e) Patagonian groups database. Square: median; box: 25%–75%; whisker: minimum-maximum values. Orange: females, blue: males. Populations showing significantly greater male fWHR (after t-test for independent samples) are marked with grey boxes.
(TIF)

Table S1 **Sample details for each database, including Pinker's sociocultural classifications.**
(XLSX)
